# The impact of dose rate optimisation and robust optimisation on FLASH proton therapy treatment plan quality and dose rates

**DOI:** 10.3389/fonc.2025.1638319

**Published:** 2025-12-19

**Authors:** Nathalie Lövgren, Rasmus Nilsson, Erik Traneus, Kristoffer Petersson

**Affiliations:** 1 Department of Oncology, University of Oxford, Oxford, United Kingdom; 2 RaySearch Laboratories AB, Stockholm, Sweden; 3 Department of Haematology, Oncology and Radiation Physics, Skåne University Hospital, Lund, Sweden

**Keywords:** FLASH proton therapy, FLASH-PT, dose rate optimisation, robust optimisation, treatment plan quality, dose rates

## Abstract

**Background and purpose:**

Bragg peak FLASH proton therapy (FLASH-PT) relies on fast dose delivery (≥ 40 Gy/s) to elicit a normal tissue sparing effect. FLASH-PT beam delivery modifications lead to inferior margin-based FLASH-PT treatment plan quality compared to intensity modulated proton therapy (IMPT). To achieve ultra-high dose rates to regions of interest, dose rate optimisation may need to be utilised as part of the treatment planning process. This study aims to determine the impact of dose rate optimisation and robust optimisation on FLASH-PT treatment plan quality and achievable dose rates. All FLASH-PT plans are also compared to IMPT plans to determine the clinical applicability of the technique.

**Materials and methods:**

FLASH-PT and IMPT treatment plans were generated for bone (*n* = 3), brain (*n* = 4) and lung (*n* = 3) targets for a one-beam-per-fraction and multi-beam-per fraction delivery, respectively. The open-source MIROpt treatment planning system (TPS) was used to generate dose rate optimised FLASH-PT plans, while a research version of the RayStation TPS was used to generate non-dose rate optimised, margin-based, and robust FLASH-PT plans. Dose rate coverage was evaluated for different dose and dose rate thresholds.

**Results and conclusion:**

Dose rate optimised FLASH-PT plans were associated with significantly worse target dose coverage, whilst significantly improving dose rate coverages to organs at risk, compared to non-dose rate optimised plans. The use of dose rate optimisation should be used with caution as it may lead to degraded plan quality. Robust optimisation improved target coverage compared to margin-based plans, without compromising dose rate coverage. FLASH-PT plans struggle to achieve IMPT-equivalent D_95%_ and is associated with non-significant increases in organ at risk doses compared to IMPT, regardless of TPSs and treatment planning techniques (margin and robust). Future work will focus on improving D_95%_, reducing organ at risk doses, and optimising MU/spot delivery to improve plan quality, while further increasing the dose rates.

## Introduction

FLASH radiotherapy (FLASH-RT), with its name attributed to the ultra-high dose rate (≥ 40 Gy/s) radiation delivery, has been shown to elicit equivalent tumour damage to conventional dose rate radiotherapy but with less normal tissue toxicity. This sparing effect is known as the “FLASH-effect” and has been observed *in vitro* for different cell lines and *in vivo* for various animal models, independent of radiation type used (electron, proton, carbon, and x-ray) (1–13).

Clinical electron beams lack the depth-penetration required for treatments of deep-seated targets, and the production of ultra-high dose rate photons is still limited to pre-clinical experiments due to vast power requirements and Bremsstrahlung target heating effects associated with the increased dose rate (14). Proton therapy is therefore the most clinically feasible modality for treatments of deep-seated targets at ultra-high dose rates, with the FAST-01 trial having demonstrated the safe use of ultra-high dose rate protons in a clinical setting (15).

To deliver Bragg peak FLASH proton therapy (FLASH-PT), conventional energy modulation cannot be adopted as the energy selection system is not fast enough to allow ultra-high dose rates to be achieved. Instead, FLASH-PT can be delivered using mono-energetic protons traversing through patient-specific energy degraders to conform the dose distribution to the target (16–20). The use of patient-specific energy modulators requires a one-beam-per-fraction delivery; single-field optimisation, along with margin-based planning, has therefore been adopted (21–24).

FLASH-PT treatment planning studies have been conducted for several different tumour types to investigate the feasibility of clinically implementing the technique and any constraints that may arise (20–23, 25–27). FLASH-PT plans are generally associated with an increase in maximum target and organ at risk (OAR) dose, as well as reduced target homogeneity, compared to standard intensity-modulated proton therapy (IMPT) plans (21, 22, 24, 27). The inferior FLASH-PT plan quality is likely due to the scattering effects of the protons interacting with the static energy degraders (mainly the range shifter). The scattering effects increase spot sizes and range straggling, negatively impacting the FLASH-PT dose distribution compared to conventional proton therapy (21, 22).

In terms of clinical use of ultra-high dose rate protons, the FAST-01 trial has been successfully completed, and the FAST-02 trial is currently ongoing, both implementing the use of transmission beam protons (15, 28, 29). Following on from this, the natural progression is to implement FLASH proton therapy using the Bragg peak to better exploit the benefits of proton therapy. An improved understanding of the feasibility and limitations of the FLASH-PT hardware (i.e., the FLASH-modified beam delivery) and software (i.e., the treatment planning system (TPS) and optimisation) is therefore crucial.

An important question to address is whether there is a need to implement dose rate objectives into the optimisation and treatment planning process. Historically, dose rates have not been of interest when generating and optimising clinical treatment plans (30). However, with the emergence of ultra-high dose rate radiotherapy, dose rate calculation and optimisation are becoming increasingly relevant, as surpassing a certain dose rate threshold could be essential for the efficacy of the treatment. Dose rate distributions should also be carefully characterised and controlled during treatment planning to determine whether sufficient ultra-high dose rate coverage to regions of interest can be achieved, and how it may impact the treatment plan quality. It is therefore important to understand the impact this additional optimisation may have on treatment plan quality.

Common dose rate definitions include the dose-averaged dose rate (DADR), percentile dose rate (DR_prct_) (also known as pencil beam scanning dose rate), instantaneous dose rate, and the average field-dose rate (31, 32). However, as no standard definition is used in the literature, varying ultra-high-dose rate coverages have been reported (21–26, 31, 33). This can become problematic if there are drastically different dose rates achieved for a patient case depending on the definition used.

With regards to treatment planning techniques, margin-based planning is the most common technique adopted for FLASH-PT; however, it is suboptimal for proton therapy due to range errors associated with the particle beam delivery (34). Instead, robust treatment planning should be implemented for FLASH-PT to make the treatment plans more robust to range uncertainties whilst also allowing for a just comparison between FLASH-PT and IMPT plans (35). Furthermore, the use of robust optimisation may also improve FLASH-PT target coverage compared to margin-based planning. Robust FLASH-PT treatment planning and its potential impact on achievable dose rates, along with direct dose comparisons to robust IMPT, have not yet been explored.

This study aims to compare FLASH-PT plans generated using two different TPSs to evaluate the impact of using dose rate objectives in the treatment planning optimisation on both the treatment plan quality and the achieved dose rates. This is, to the knowledge of the authors, a novel comparison which has not yet been shown in the literature. Another novel comparison, with respect to FLASH-PT, is that of margin-based versus robust FLASH-PT treatment planning, to investigate the impact of robust optimisation on FLASH-PT dose and dose rate coverage (both DR_prct_ and DADR). FLASH-PT plans are also compared to IMPT plans, for each TPS and each optimisation technique (margin and robust), to determine the clinical applicability of Bragg peak FLASH proton therapy.

## Materials and methods

A research version of RayStation^®^ (RaySearch Laboratories AB, Stockholm, Sweden), and the open-source MIROpt TPS, along with the ConformalFLASH library (www.openFLASH.software), accessed through OpenREGGUI, were used to simulate all treatment plans (36–38). The comparison between an open-source TPS (MIROpt) to a research version of a clinically validated system (RayStation) is due to the limited availability of TPSs such as MIROpt which are able to generate dose rate optimised FLASH-PT treatment plans. FLASH-PT and IMPT plans were generated for bone (*n* = 3), brain (*n* = 4), and lung (*n* = 3) targets; patient information is outlined in **Table 1**. The same patient cases were used in a previous study by Lövgren et al. (2024), where the feasibility and constraints of FLASH-PT treatment planning using the MIROpt TPS, along with the ConformalFLASH library, was investigated (21).

**Table 1:** Patient information outlining the target type (bone, brain, lung), target volume (cm^3^), prescribed dose (Gy [RBE]), number of fractions, and dose per fraction (Gy [RBE]) given in terms of the relative biological effective (RBE) dose.

Patient case	Target volume (cm^3^)	Prescribed dose (Gy [RBE])	Number of fractions	Dose per fraction (Gy [RBE])
Bone 1 (vertebrae)	61.78	8	1	8
Bone 2 (vertebrae)	323.53	8	1	8
Bone 3 (vertebrae)	206.86	8	1	8
Brain 1	191.65	20	2	10
Brain 2	0.20	30	3	10
Brain 3	0.46	30	3	10
Brain 4	12.45	34	2	17
Lung 1	11.78	45	3	15
Lung 2	10.58	45	3	15
Lung 3	21.47	45	3	15

IMPT plans were generated using a conventional beam setup and multi-field optimisation (MFO). The FLASH-PT beam setup includes monoenergetic (230 MeV) pencil beam scanned protons, scanned across a conformal energy modulator, range shifter, and an aperture block. Plans were generated for use on the Ion Beam Applications Proteus^®^Plus (Louvain-la-Neuve, Belgium) machine, with a maximum field size of 8 cm x 8 cm x 8 cm for the MIROpt TPS and 10 cm x 10 cm x 8.4 cm for the RayStation TPS. Beam currents were 300 nA and 500 nA for the highest energy of IMPT and FLASH-PT, respectively.

For each patient case, the number of fields and beam angles used were the same for both IMPT and FLASH-PT plans. Due to the patient-specific beam setup, all FLASH-PT treatment plans were generated for a one-beam-per-fraction delivery along with single-field-uniform-dose optimisation (SFUDO). Fractionation schemes are outlined in **Table 1**. To compare FLASH-PT to IMPT, the individual fraction FLASH-PT plans were summed such that the total dose distributions were compared. A relative biological effectiveness of 1.1 was assumed for all IMPT and FLASH-PT plans.

Robust optimisation was not available for FLASH-PT in the MIROpt TPS; margin-based planning was therefore adopted for all MIROpt plans. To better understand the impact of dose rate optimisation on the plan quality and achievable dose rates, margin-based plans were also generated using RayStation. The planning target volume (PTV) was used to generate all margin-based plans. The dose distributions were evaluated on the clinical target volume (CTV), except for Brain 2, Brain 3, and Brain 4 where no CTV was available and so the gross tumour volume (GTV) was utilised instead.

Robust optimisation was available in RayStation and was used to generate additional FLASH-PT and IMPT plans. Set-up uncertainties were implemented as the difference between CTV (or GTV) and PTV, along with density uncertainties of 3.5% for the bone and brain cases, and 4.5% for the lung cases. The nominal (non-shifted) scenario was considered for the plan evaluation. Additionally, the worst-case scenario was evaluated for D_95%_ (%) and D_2%_ (%) for both margin-based and robust treatment plans. Any subsequent reference to target dose, for any treatment plan, refers to dose to the CTV (and GTV for Brain 2, Brain 3, and Brain 4) in the nominal scenario unless the worst-case scenario is explicitly stated. All treatment plans were normalised such that the median dose to the target was the prescribed dose. The optimisation objectives used for an example brain case, for FLASH-PT plans simulated using both MIROpt and RayStation (margin and robust), can be found in the Supplementary Materials (lines 1–80 for MIROpt and **Supplementary Figures S1-S2** for RayStation).

FLASH-modified dose distributions have shown large variations in the potential OAR sparing depending on the tumour location, with some FLASH-RT scenarios even leading to no net sparing (39). Therefore, if the FLASH-PT plans can be as similar to IMPT plans as possible, any sparing effect that may be elicited will be an additional benefit to the patient. Consequently, this study investigates FLASH-PT plan quality without accounting for a sparing effect.

Dose rates were calculated using both the DR_prct_ (95^th^ and 98^th^ percentile) and the DADR definitions. The DR_prct_ for a voxel *i*, is defined by Folkerts et al. (32) as:

(1) $$\begin{array}{c} {\text{DR}}_{\text{prct}}=\frac{D(i)-2d_{thr}(i,\;t_{0})}{t_{1}-t_{0}} \end{array}$$

where D(i) is the total prescribed dose (100%), $d_{thr}(i,\;t_{0})$ (%) is a minimum dose threshold given as a percentage of the total dose, $t_{0}$ is the time at which the dose threshold at *i* is exceeded, and $t_{1}$ is the time at which the accumulated dose $d(i,\;t_{1})=D(i)-d_{thr}(i,t_{0})$ (32). The DADR definition does not explicitly incorporate dose delivery time and is defined by van de Water et al. (31), for a voxel *i*, as:

(2) $$\begin{array}{c} {\text{DADR}}_{i}=\;\sum_{j=1}^{n}\frac{\left(d_{ij}w_{j})(d_{ij}BI_{j}\right)}{\sum_{j=1}^{n}d_{ij}w_{j}} \end{array}$$

where *j* represents a spot, *w* is the associated spot weight, *d* is the dose-influence matrix, and *BI* is the beam intensity (31).

Dose rates were optimised using a dose rate objective function. Input parameters in this objective function include: a minimum dose to the OAR (D_thr_ (Gy)) at which the dose rate is evaluated, a reference dose rate (DR_thr_ (Gy/s)), and an importance weight for the function. An example of the implementation of such a dose rate objective is shown in **Supplementary Materials** on lines 51-59. Dose rates were optimised using the following approach: for a given OAR structure, the dose rate is evaluated on a voxel-by-voxel level and then averaged across all voxels receiving a dose ≥ D_thr_. If the averaged dose rate (DR) is less than DR_thr_, a penalty proportional to (DR – DR_thr_)^2^ is added to the objective function (37). A minimum dose threshold of D_thr_ = 2 Gy (total dose) and a reference dose rate of DR_thr_ = 40 Gy/s were used in the dose rate objective function (evaluated for the 98^th^ percentile) for all the OARs receiving a non-zero dose in the MIROpt FLASH-PT plans. Low importance weights for the dose rate objectives ( ~10^-8^ on a scale of 0-1) were used as the dose rate optimisation would otherwise dominate the treatment plan optimisation, severely degrading the plan quality.

In MIROpt, the DR_prct_ and DADR were directly calculated as part of the treatment planning process using the ConformalFLASH library. In RayStation, DR_prct_ and DADR calculations were computed using separate dose rate scripts working alongside the TPS. Dose rate improvements in RayStation involved the removal of low-weighted spots (spots with MU less than 20 MU/fx), unless the plan quality was severely impacted.

In this study, dose rate coverage was defined as the relative number of non-target voxels receiving total doses ≥x Gy at dose rates ≥y Gy/s, where x ={2, 4, 6, 8} and y={40, 70, 100}. Dose rate coverages were evaluated for each organ at risk within each individual fraction, and coverages below 1% were excluded as values below this limit were smaller than the associated uncertainty of the simulated data.

Treatment plan comparisons were made using dose metrics. Minimum and maximum target dose limits were set as D_95%_ = 95% and D_2%_ = 105%, respectively. Target dose homogeneity was evaluated using (D_2%_-D_98%_)/D_50%_. OAR dose protocols used were RTOG 0631 (bone), CORSAIR (brain and lung), and the UK Consensus on Normal Tissue Dose Constraints for Stereotactic Radiotherapy (lung), detailed information can be found in the **Supplementary Materials (Tables S2-S7)** (40–42). Plans were optimised with the aim of the OAR doses adhering to the OAR dose protocols where possible. Statistical comparisons were performed using the two-sided Wilcoxon Rank Sum Test, with significance defined as *p* < 0.05.

FLASH-PT plans generated using MIROpt and RayStation will be referred to as dose rate optimised (DRO) and non-dose rate optimised (non-DRO) plans, respectively.

## Results

For the inter-system comparisons, Bone 1, Bone 2, Bone 3, and Brain 1 were excluded due to target size constraints using the MIROpt TPS; this was imposed by the field size which limited the space available for the conformal energy modulator in the nozzle.

**Figure 1:**
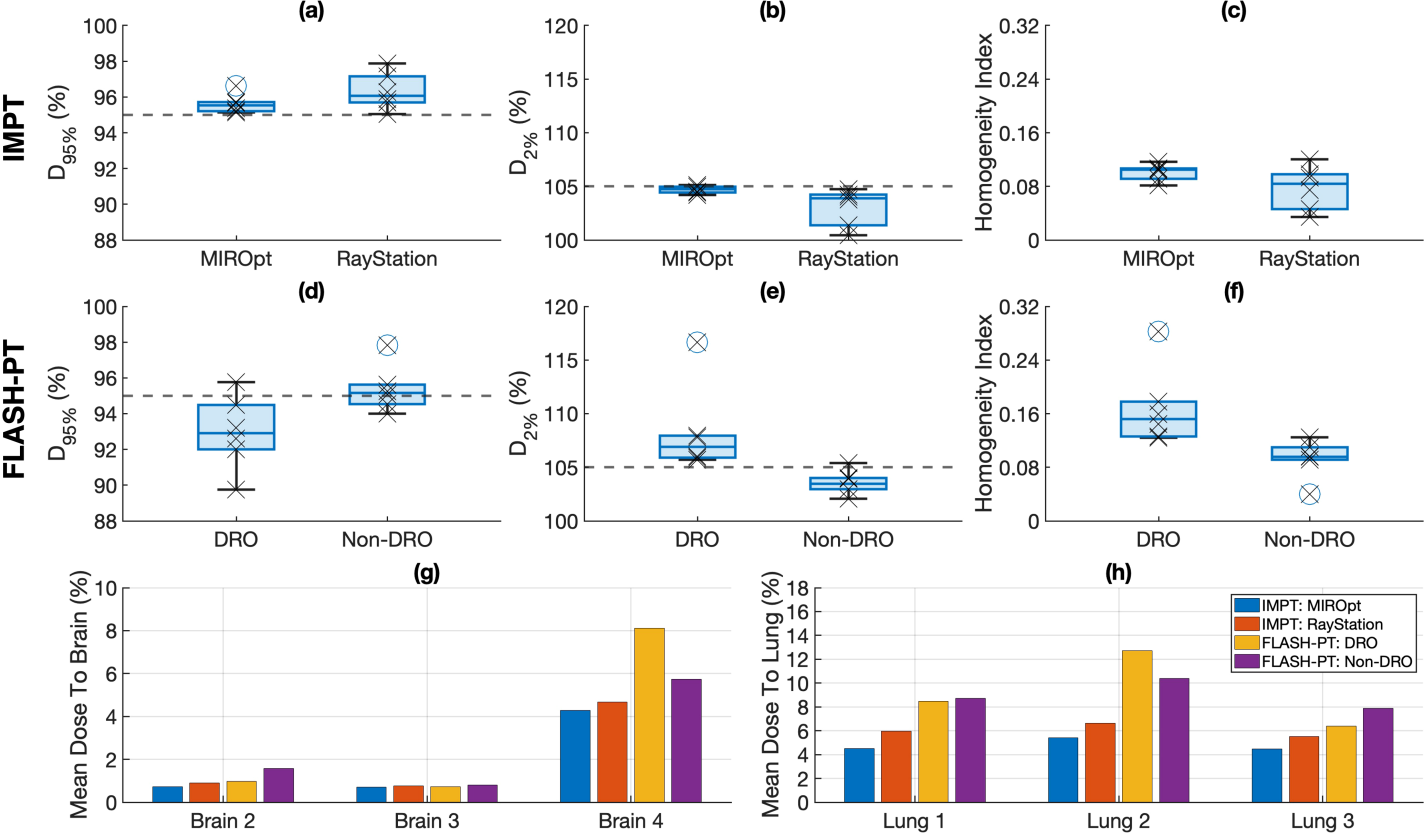
Target and organ at risk dose comparisons for margin-based plans for the different treatment planning systems (TPSs) (MIROpt vs. RayStation) and optimisation techniques (dose rate optimised (DRO) and non-dose rate optimised (non-DRO)). Boxplots comparing **(a)** D_95%_ (%), **(b)** D_2%_ (%), and **(c)** the homogeneity index (HI) ((D_2%_ (%) – D_98%_ (%))/D_50%_ (%)) for IMPT plans generated using MIROpt and RayStation are shown in the top panel. The middle panel shows boxplots comparing **(d)** D_95%_ (%), **(e)** D_2%_ (%), and **(f)** the HI for DRO and non-DRO FLASH-PT plans. Each point (x) shows the exact D_95%_ (%), D_2%_ (%), and HI for each patient case. The line within the box represents the median value, the bottom and top of the box represent the 25^th^ and 75^th^ percentiles, the whiskers show the range of the data points (excluding outliers), and outliers are shown using individual (o) markers. Grey dashed lines indicate target dose limits of **(a)** D_95%_ = 95% and **(b)** D_2%_ = 105%. Mean doses to the **(g)** brain structure and **(h)** (irradiated) lung structure for the different TPSs and treatment techniques are represented using bar charts. All doses are normalised with respect to the prescribed dose.

The minimum target dose limit was not achieved for all margin-based FLASH-PT plans, irrespective of TPS. No significant differences in D_95%_ were found between the DRO and non-DRO FLASH-PT plans, or between the IMPT and FLASH-PT plans within each TPS (**Figure 1a, d**). The non-DRO FLASH-PT plans were associated with significantly lower target D_2%_ compared to the DRO plans (**Figure 1e**), with RayStation improving D_2%_ for the IMPT plans compared to MIROpt (**Figure 1b**). FLASH-PT target homogeneity was also significantly improved for non-DRO plans (**Figure 1f**). IMPT target homogeneity was similar across the TPSs (**Figure 1c**).

All margin-based FLASH-PT plans (both DRO and non-DRO), except for the DRO FLASH-PT plan for Lung 3, adhered to the mandatory OAR dose constraints (**Supplementary Materials, Tables S2-S7**). No significant differences in mean dose to the brain were found between the TPSs for IMPT, and dose rate optimisation techniques (DRO vs. non-DRO) (**Figure 1g**). Despite a trend of increased mean dose to the lung for the FLASH-PT plans compared to that of IMPT, there were no significant differences between the systems or dose rate optimisation techniques used (**Figure 1h**). Compared to IMPT, FLASH-PT plans were associated with non-significant increases in OAR doses and reduced dose conformity using both TPSs (**Figures 1g, h**).

**Figure 2:**
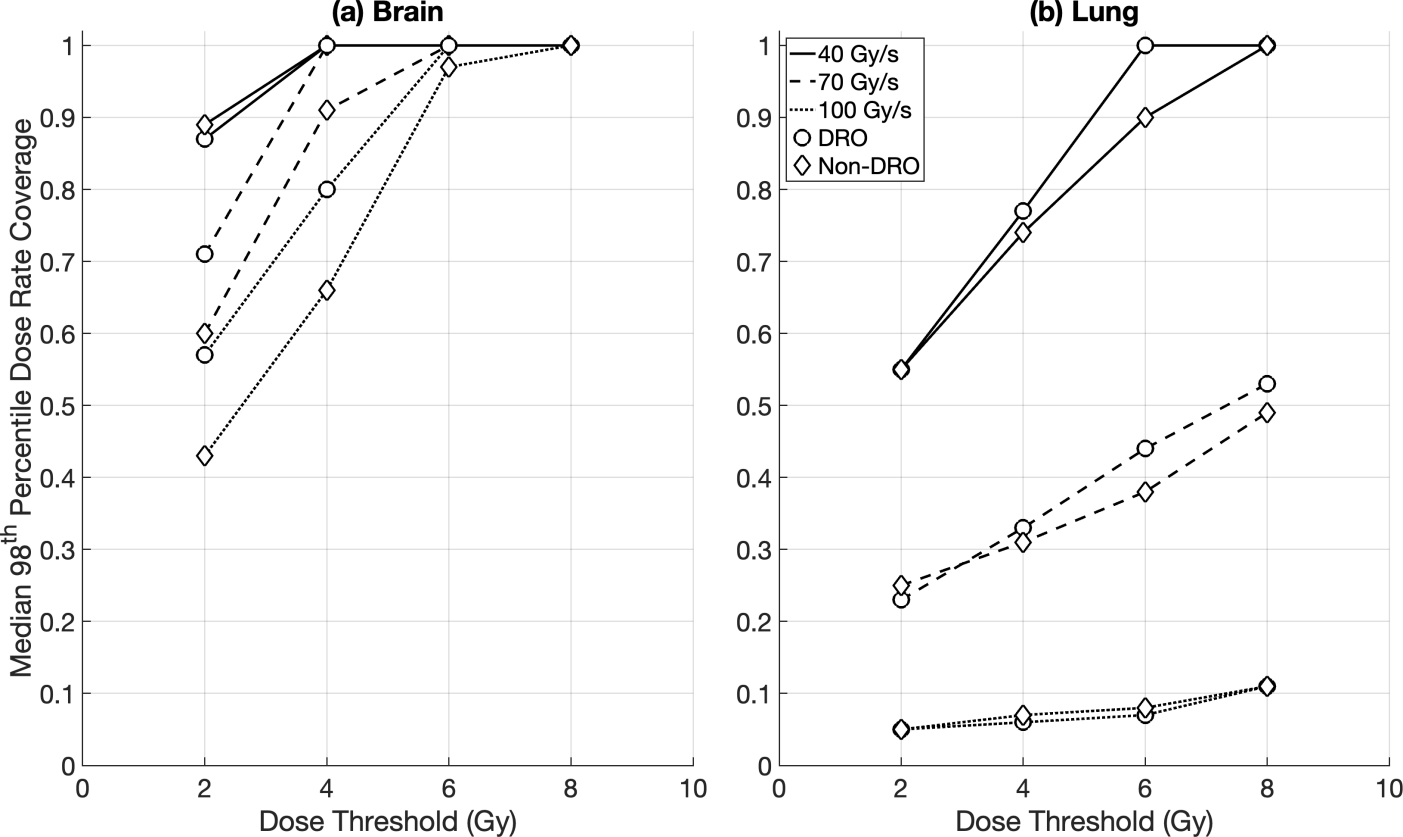
Median percentile dose rate (98^th^ DR_prct_) coverage for margin-based plans for **(a)** brain and **(b)** lung cases for the different optimisation techniques: dose rate optimised (DRO) (circle), and non-dose rate optimised (non-DRO) (diamond). Dose rate coverages were evaluated for all organs at risk using dose thresholds of 2 Gy, 4 Gy, 6 Gy, and 8 Gy, and dose rate thresholds of 40 Gy/s, 70 Gy/s, and 100 Gy/s.

**Figure 3:**
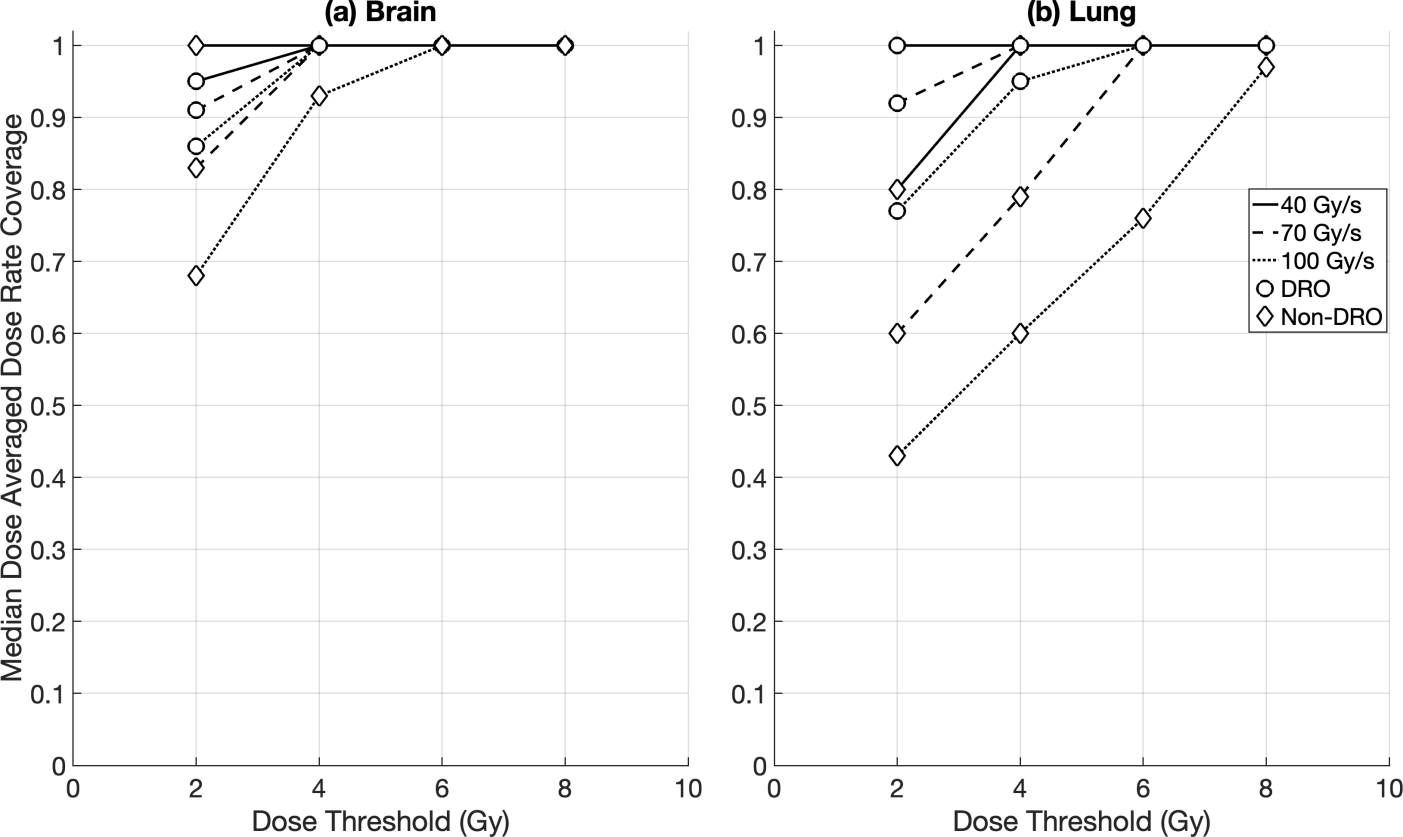
Median dose averaged dose rate (DADR) coverage for margin-based plans for **(a)** brain and **(b)** lung cases for the different optimisation techniques: dose rate optimised (DRO) (circle), and non-dose rate optimised (non-DRO) (diamond). Dose rate coverages were evaluated for all organs at risk using dose thresholds of 2 Gy, 4 Gy, 6 Gy, and 8 Gy, and dose rate thresholds of 40 Gy/s, 70 Gy/s, and 100 Gy/s.

Intra-fraction DR_prct_ coverages for the brain OARs were similar between the DRO and non-DRO plans, except for DR_thr_ = 70 Gy/s where the use of dose rate optimisation achieved a significantly higher coverage (median ≥ 71%) compared to plans with no dose rate optimisation (median ≥ 60%) (**Figure 2a**). Non-DRO FLASH-PT plans achieved significantly lower DR_prct_ coverages for the lung cases for DR_thr_ = 40 Gy/s compared to DRO plans (although both had median coverages ≥ 55%), whereas no significant differences were found at the two higher dose rate thresholds (**Figure 2b**). For the DADR coverages, DRO plans outperformed the non-DRO plans for all dose and dose rate thresholds, and for both brain and lung targets, except for DR_thr_ = 40 Gy/s for the brain cases where DRO plans and non-DRO plans achieved median coverages of ≥ 95% and 100%, respectively (**Figure 3**).

**Figure 4:**
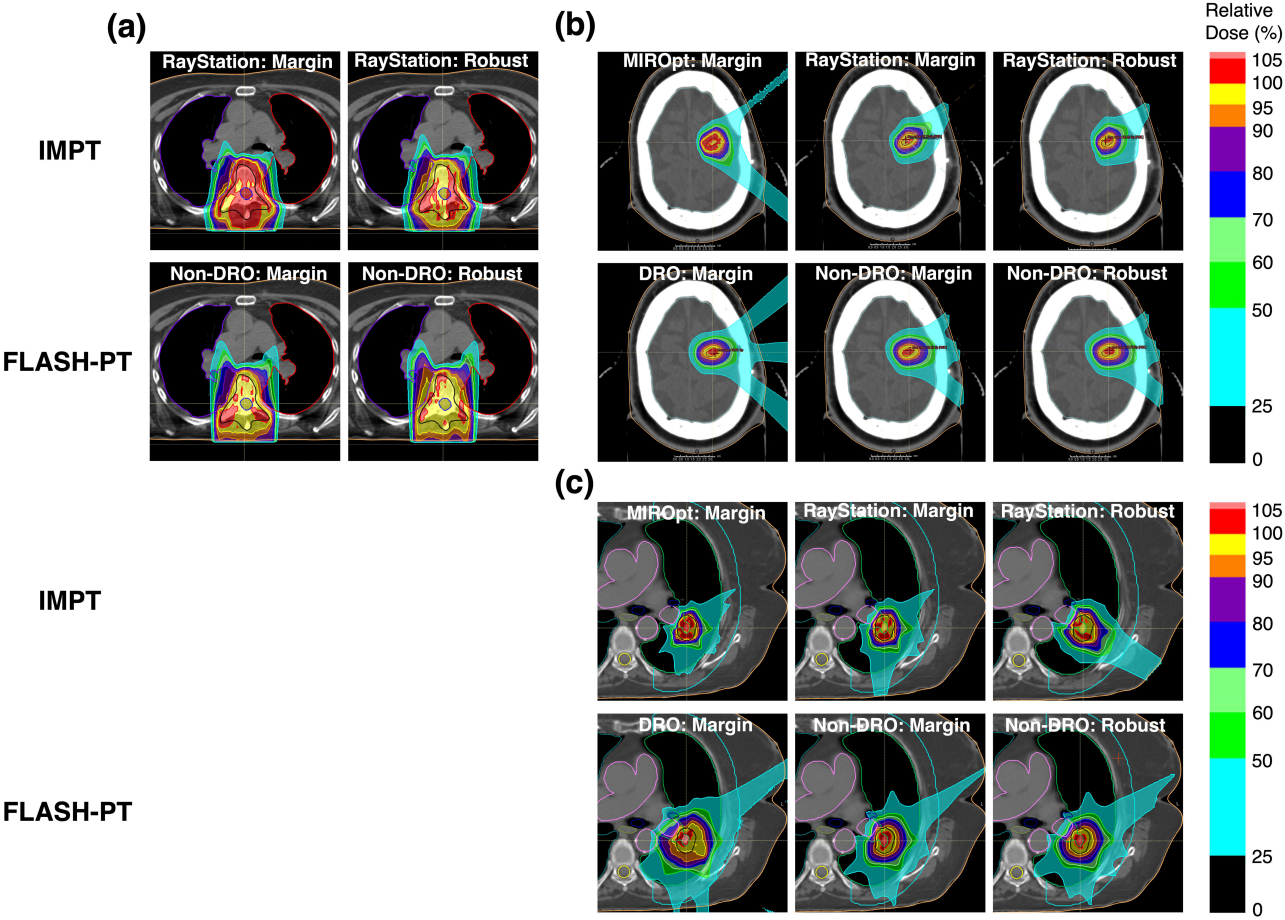
Dose map comparisons of example patient cases for the **(a)** bone, **(b)** brain, and **(c)** lung targets using the different treatment planning systems (MIROpt and RayStation) for the IMPT plans, optimisation techniques (dose rate optimised (DRO) and non-dose rate optimised (non-DRO)) for the FLASH-PT plans, and treatment planning techniques (margin-based and robust optimisation) for both FLASH-PT and IMPT plans. Doses are normalised with respect to the prescribed dose. Plans could not be generated for the bone cases in MIROpt due to target size constraints. The specific cases considered are Bone 1, Brain 2, and Lung 2.

Large margins for spot placement had to be used for the DRO FLASH-PT lung plans to achieve a reasonably high D_95%_ (≥ 85%); this drastically reduced the DRO FLASH-PT dose conformality in comparison to that achieved for the corresponding non-DRO plans (**Figure 4**). FLASH-PT plans were associated with reduced dose conformity compared to IMPT, irrespective of treatment planning technique (margin-based vs. robust) (**Figure 4**). The reduction in conformity, however, was not as prominent for targets located in areas with fewer density differences (e.g., bone and brain) compared to the lung targets (**Figure 4**).

The increased field size allowed by RayStation reduced the target size constraint previously imposed by the MIROpt TPS. All patient cases were therefore included in the evaluations and comparisons between margin-based plans and plans generated using robust optimisation; this led to margin-based FLASH-PT RayStation plans achieving a median D_95%_ ≥ 95%. Still, on an individual-case basis, several margin-based FLASH-PT plans (Bone 2, Lung 1, and Lung 3) did not fulfil the target dose constraints.

**Figure 5:**
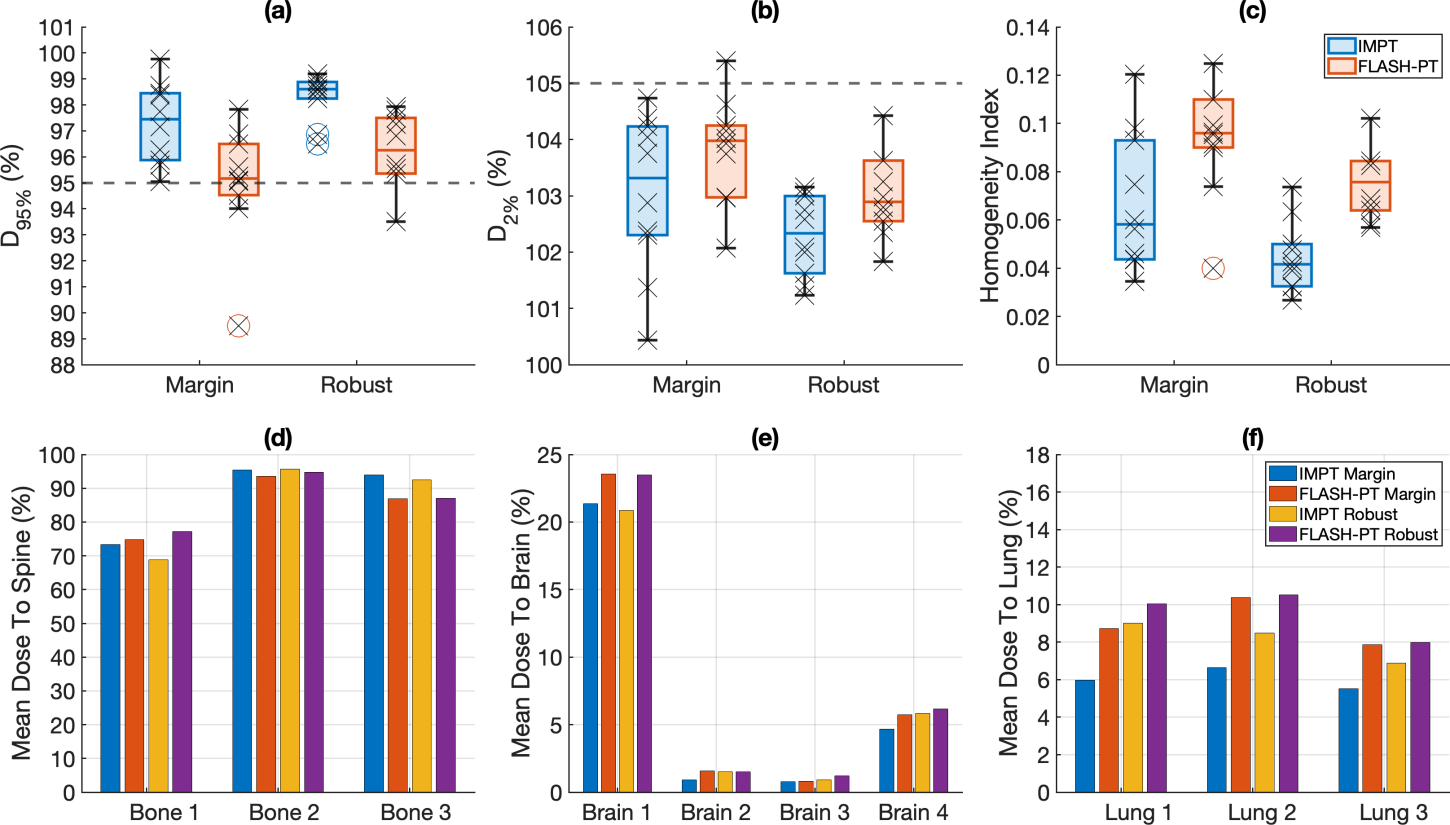
Target and organ at risk dose comparisons for the different treatment planning techniques (margin-based vs. robust optimisation). Boxplots comparing **(a)** D_95%_ (%), **(b)** D_2%_ (%), and **(c)** the homogeneity index (HI) ((D_2%_ (%) – D_98%_ (%))/D_50%_ (%)) for each treatment planning technique and each treatment technique (IMPT (blue) and FLASH-PT (red)). Each point (x) shows the exact D_95%_ (%), D_2%_ (%), and HI for each patient case. The line within the box represents the median value, the bottom and top of the box represents the 25^th^ and 75^th^ percentiles, the whiskers show the range of the data points (excluding outliers), and outliers are shown using individual (o) markers. Grey dashed lines indicate target dose limits of **(a)** D_95%_ = 95% and **(b)** D_2%_ = 105%. Mean doses to the **(d)** spine, **(e)** brain structure and **(f)** (irradiated) lung structure for the different planning techniques and treatment techniques are represented using bar charts. All doses are normalised with respect to the prescribed dose.

The use of robust optimisation improved D_95%_ for both FLASH-PT and IMPT plans compared to the margin-based plans in both the nominal and worst-case scenario (**Figure 5a**). All robust FLASH-PT plans, except for Bone 2, adhered to the target dose limits; the width (inline and crossline) of the Bone 2 target was greater than the maximum field size used for the FLASH-PT modified beam setup. Robust optimisation produced FLASH-PT and IMPT plans with reduced D_2%_ (nominal and worst-case) and improved target homogeneity compared to their margin-based counterparts (**Figures 5b, c**). There were non-significant differences in the nominal scenarios between margin-based and robust plans for both FLASH-PT and IMPT, except for the IMPT homogeneity index which was significantly improved when using robust optimisation. Comparisons between robust IMPT and FLASH-PT yielded significant differences in D_95%_ and the homogeneity index (**Figure 5a, c**).

Robust FLASH-PT significantly improved the worst-case scenarios for both D_95%_ and D_2%_ compared to the margin based worst-case scenarios (**Supplementary materials, Figure S3** and **Supplementary Table S1**). Unlike IMPT, FLASH-PT plans could not adhere to both target dose constraints for the worst-case scenarios when aiming to minimise doses to OARs such that they adhered to the OAR dose protocols. FLASH-PT achieved significantly lower worst-case D_95%_ compared to IMPT with D_95%_ = 90.64% compared to D_95%_ = 95.33%, respectively (**Supplementary Figure S3**). However, the majority of worst-case FLASH-PT plans still adhered to D_2%_ ≤ 105%.

Similar to the margin-based FLASH-PT plans, robust FLASH-PT plans were also associated with non-significant increases in OAR doses compared to IMPT, with the lung cases showing the largest increase. The same trend was seen when comparing margin-based planning to robust optimisation for both IMPT and FLASH-PT (**Figures 5d-f**). All margin, and robust, IMPT and FLASH-PT plans generated using RayStation adhered to the OAR dose constraints (**Supplementary Materials, Tables S2-S7**).

**Figure 6:**
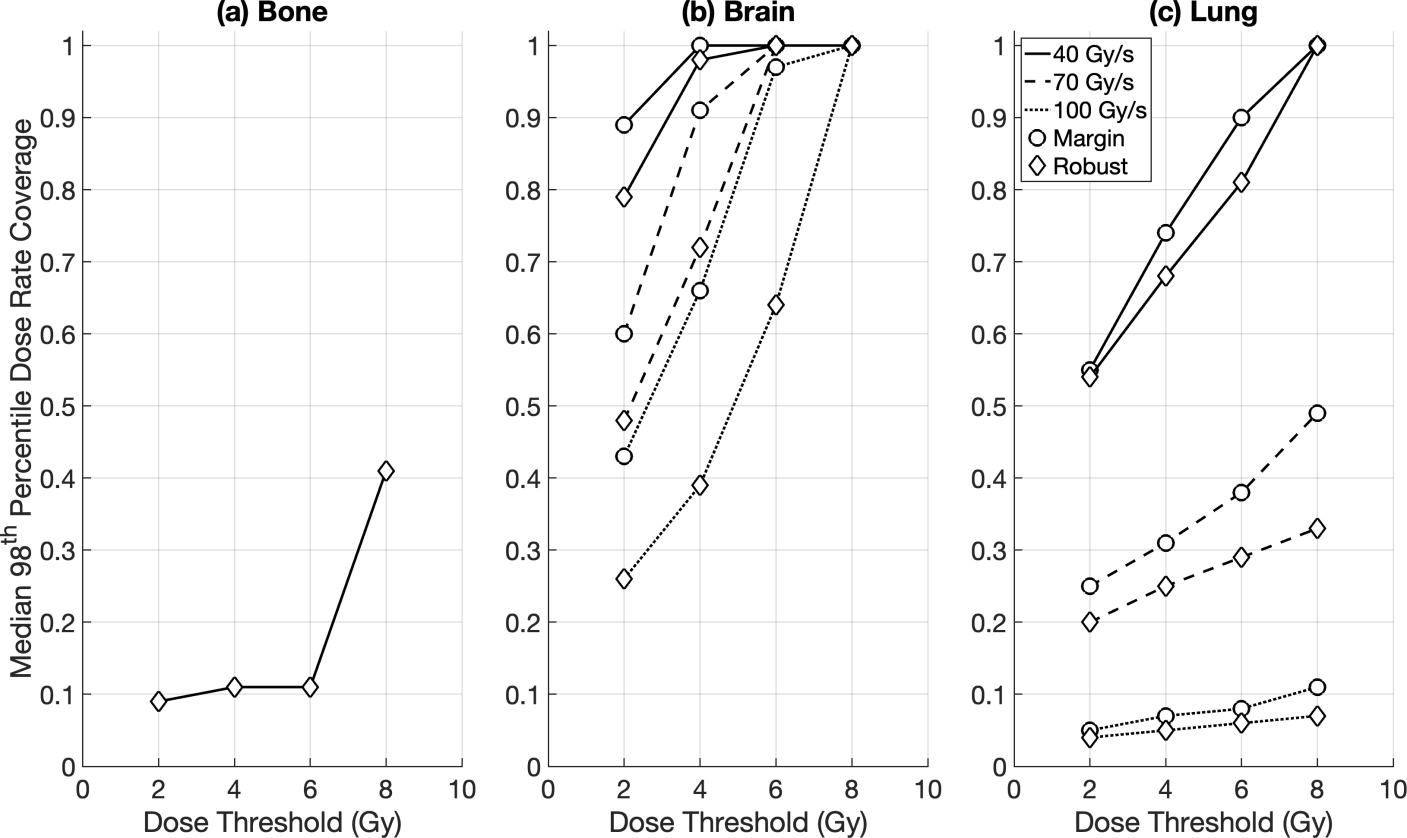
Median percentile dose rate (98^th^ DR_prct_) coverage for RayStation plans for **(a)** bone, **(b)** brain, and **(c)** lung cases for the different treatment planning optimisation techniques: margin-based (circle), and robust (diamond). Dose rate coverages were evaluated for all organs at risk using dose thresholds of 2 Gy, 4 Gy, 6 Gy, and 8 Gy, and dose rate thresholds of 40 Gy/s, 70 Gy/s, and 100 Gy/s. No dose rate coverage was achieved for margin-based bone plans for any dose or dose rate threshold, or for the robust bone plans at dose rate thresholds of 70 Gy/s and 100 Gy/s.

**Figure 7:**
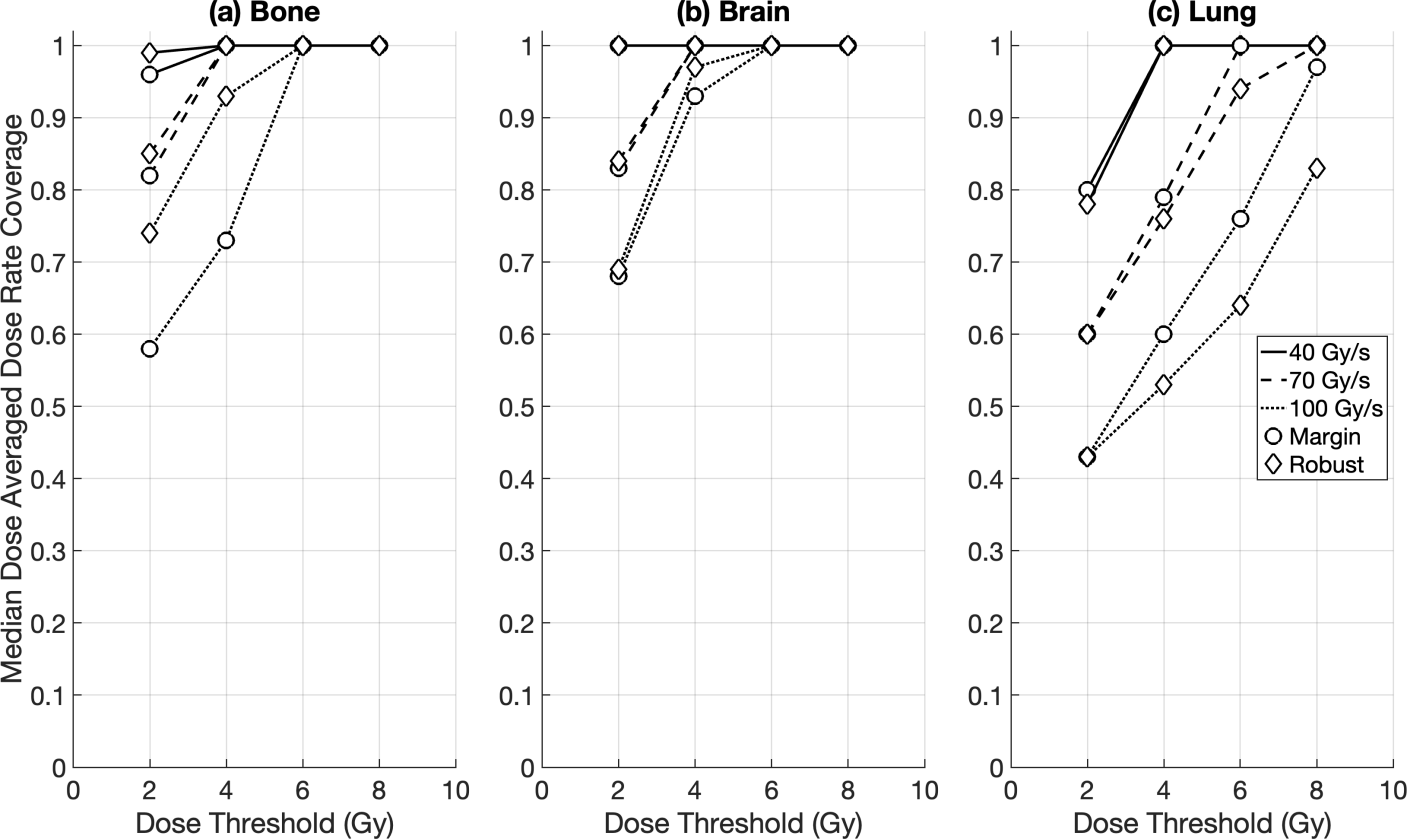
Median dose averaged dose rate (DADR) coverage for RayStation plans for **(a)** bone, **(b)** brain, and **(c)** lung cases for the different treatment planning optimisation techniques: margin-based (circle), and robust (diamond). Dose rate coverages were evaluated for all organs at risk using dose thresholds of 2 Gy, 4 Gy, 6 Gy, and 8 Gy, and dose rate thresholds of 40 Gy/s, 70 Gy/s, and 100 Gy/s.

DR_prct_ coverage was not achieved for the margin-based bone plans for any dose or dose rate threshold, whereas minimal coverage was found for the robust bone plans for DR_thr_ = 40 Gy/s (**Figure 6a**). Consequently, no statistical DR_prct_ comparison could be made between the margin-based and robust treatment plans for the bone targets. For the remaining cases, the differences between margin-based and robust DR_prct_ were non-significant, except for DR_prct_ = 100 Gy/s and DR_prct_ = 70 Gy/s for the brain and lung cases, respectively (**Figure 6b, c**). No major difference was found between the 95^th^ and 98^th^ DR_prct_ definitions across all patient cases and optimisation techniques (margin vs. robust). DADR coverages were significantly higher than the DR_prct_ coverages for all cases and optimisation techniques. Similar DADR coverages were achieved for margin-based and robust FLASH-PT plans for each dose rate threshold and target type (**Figure 7**).

Dose volume histograms for the different TPSs, treatment techniques, and treatment planning techniques for the example patient cases shown in **Figure 4** are included in the **Supplementary Materials (Figures S4-S6)**.

## Discussion

No sparing effect was accounted for in the FLASH-PT treatment planning process. The results in this study therefore only represent the feasibility and limitations of the dose distributions achieved using the FLASH-modified beam setup when compared to IMPT.

Non-DRO FLASH-PT plans were associated with significant improvements in target dose coverage (D_2%_ and homogeneity) compared to DRO plans, with non-significant differences in OAR doses between the optimisation techniques, despite the non-DRO FLASH-PT plans showing a trend of reduced OAR doses. DRO FLASH-PT plans consistently achieved higher dose rate coverages than non-DRO plans across dose thresholds, dose rate thresholds, target types, and dose rate definitions. This was particularly exemplified for the brain cases, where the DRO plans maintained higher DR_prct_ coverages at the higher dose rate thresholds (≥ 70 Gy/s) across most dose levels, indicating a greater ability to sustain high-dose rate conditions compared to the non-DRO plans. For the lung cases, a clear discrepancy was observed: margin-based non-DRO plans produced significantly lower DR_prct_ at 40 Gy/s, whereas DRO plans were able to maintain higher coverages despite the challenges of tissue heterogeneity. The higher dose rate coverages associated with the DRO plans were also seen for the DADR definition.

Regions of greater density differences (such as lungs), where it is inherently more difficult to achieve high dose rate coverages, may require dose rate optimisation compared to regions with fewer density differences (such as the brain). However, the large variation in dose rate coverages along with the difficulty in controlling the dose rates for the lung cases (for the different TPSs, treatment planning techniques, and dose rate definitions) serve as an indication of the lungs being a poor FLASH-PT site candidate.

A clinical observation by Daartz et al. (2024) found that areas of elevated (sub-FLASH) instantaneous dose rates correlated to regions of necrosis in a brain patient who had received proton therapy (30). Areas of elevated dose rates also receive more dose, and as the area of necrosis was distal to the target, it is not implausible that it could also be correlated to an increase in linear energy transfer and relative biological effectiveness (43). However, if sub-FLASH instantaneous dose rates do cause normal tissue damage, the use of a TPS which utilises a dose rate optimiser may be of greater interest to minimise the prevalence of sub-FLASH dose rate hotspots in normal tissues. Conversely, *in vivo* studies by Paillas et al. (2025) and Singers Sørensen et al. (2024) found reduced normal tissues toxicities at dose rates as low as 1 Gy/s and 2 Gy/s for single dose deliveries, respectively (44, 45). Both studies also showed an increase in the normal tissue sparing effect with increasing dose rate. So, if the FLASH effect can be elicited for a range of dose rates (given increased fraction doses), with an effect proportional to the dose rate, a dose rate optimising TPS may not be essential, and a TPS which improves target and OAR doses may be of more benefit. While the studies by Daartz et al., Paillas et al., and Singers Sørensen et al. differ in many aspects, they still highlight the need for further investigations on the impact of dose rates below the 40 Gy/s threshold, and the corresponding fraction doses, on normal tissue toxicity.

With respect to dose rate coverages achieved, DRO FLASH-PT plans outperformed the non-DRO plans, which is not unexpected. Nevertheless, target and OAR doses remain the top priority, and MIROpt was also associated with worsened FLASH-PT plan quality compared to RayStation.

Given the low importance weight required for the MIROpt dose rate objective function to not dominate over the treatment plan optimisation, the inclusion of the dose rate optimiser could be one of the reasons for the reduction in plan quality. Once a similar dose rate optimisation function has been implemented in RayStation, a future comparison between the two systems may offer more insight; until then, dose rate optimisation which directly impacts the dose optimisation should be used with caution, as it may significantly degrade treatment plan quality. However, different strategies have been shown to increase the dose rate without explicitly being part of the dose calculation and optimisation, with examples including: a sequential scan-pattern optimisation proposed by José Santo et al. (2023), a fast spot order demonstrated by Wase et al. (2025), a spot delivery sequence optimisation demonstrated by Zhao et al. (2024), and a spot-position and spot-weight optimisation proposed by Lansonneur et al. (2024) (46–49).

Robust FLASH proton therapy has only been investigated in the context of comparing Bragg peak to transmission beam treatment plans, with other studies comparing robust IMPT to margin-based FLASH-PT (19, 22, 23, 25). The results in this study therefore provide important insight into the feasibility and limitations of robust and margin-based FLASH-PT by directly comparing the FLASH-PT plans to their conventional IMPT counterparts. Robust FLASH-PT plans yielded significantly better target dose coverage in the worst-case scenarios than the corresponding margin-based plans. Despite robust FLASH-PT plans trending towards improved target dose coverage in the nominal scenarios, the differences when compared to margin-based plans were non-significant. The improvement may become statistically significant if each target type is investigated individually with larger sample sizes.

DR_prct_ and DADR coverages were generally unaffected by the treatment planning technique used, except for the DR_prct_ coverages at DR_thr_ = 100 Gy/s and DR_thr_ = 70 Gy/s for the brain and lung cases, respectively. These thresholds may indicate a limit at which margin-based and robust planning begin to diverge, with brain cases having a higher threshold than lungs. However, as the DR_prct_ coverage for the lung cases at DR_thr_ = 100 Gy/s (for the two techniques) converged again, the behaviour may not be so straightforward. Nevertheless, the benefits of improved target dose coverage associated with robust FLASH-PT planning can be exploited without sacrificing ultra-high dose rate coverage, potentially up to a certain limit depending on target location.

In terms of clinical applicability, however, robust FLASH-PT plans still do not match target dose coverage, doses to OARs, and dose conformality when compared to their IMPT counterparts. This could partly be due to the use of SFUDO for the FLASH-PT plans compared to the use of MFO for IMPT. However, for the exact same patient cases considered in this study, Lövgren et al. (2024) found no significant differences in target dose coverage when comparing MFO to SFUDO for margin-based IMPT plans generated using MIROpt (21). As the discrepancy between the FLASH-PT and IMPT plans extend across TPSs and treatment planning techniques, the difference in treatment plan quality is more likely due to the FLASH-modified beam setup which impose scattering effects and range straggling. The scattering effects lead to increased spot sizes, making it more difficult to achieve an equivalent dose distribution between the two techniques.

The choice of dose rate definition used in studies is critical given the variations in dose rate coverages reported in the literature, and as shown in this study (21–26, 31, 33). The use of the DADR definition significantly increased the dose rate coverages compared to the DR_prct_ definition, regardless of TPS, treatment planning technique (margin vs. robust), or target type (bone, brain, lung). The impact of this substantial increase is particularly exemplified by the difference in dose rate coverage for the bone cases. The use of the DR_prct_ definition warranted exclusion of the bone cases from the study as the dose rate coverage was too low for the FLASH sparing effect to be of benefit to the patient. However, the DADR definition would favour FLASH-PT for the patient cases due to its potential dose rate coverage of ≥ 80% for dose rates of 40 Gy/s and 70 Gy/s. The same trend was observed for the lung cases. The significant increase in dose rate coverage is likely a by-product of the DADR formulation (Equation 2), where the cumulative effect of all the low-dose voxels amounts to a higher DADR without explicitly incorporating the irradiation time. In contrast, the DR_prct_ definition, which depends on the irradiation time (Equation 1), leads to more variations in dose rate coverages; these variations appear dependent on the dose threshold, dose rate threshold, and target location. Multiple dose rate definitions should therefore be incorporated in treatment planning studies to ensure that the data is fairly represented.

Robust optimisation enhanced target dose coverage and improved FLASH-PT and IMPT treatment plans irrespective of target location, significantly improving the worst-case scenarios for both treatment techniques compared to margin-based planning. Nonetheless, the majority of FLASH-PT plans did not achieve D_95%_ ≥ 95% in the worst-case scenarios; D_95%_ therefore needs to be improved, and doses to OARs still need to be reduced, before the clinical implementation of FLASH-PT can be realised. The reduction in scattering effects and range straggling, due to the FLASH-PT modified beam delivery, will be key in generating IMPT-equivalent FLASH-PT treatment plans. It would be of interest to investigate how these effects can be reduced whilst maintaining ultra-high dose rates. A potential avenue to explore is reducing the initial energy of the FLASH-PT beam; this would reduce the amount of material needed in the energy modulator, thereby reducing scattering and range straggling. Further to this, if the energy selection process can occur more rapidly through the use of a superconducting gantry, as proposed by Zeng et al., the static energy modulators will become redundant, and the scattering effects reduced (23). If either solution can be realised, Bragg peak FLASH proton therapy will be one step closer to clinical implementation.

## Conclusion

This study aimed to investigate the impact of dose rate optimisation and robust optimisation on FLASH-PT treatment plan quality and dose rates. Comparisons between FLASH-PT and IMPT plans were also made to determine the clinical applicability of Bragg peak FLASH proton therapy, without accounting for a sparing effect.

The use of dose rate optimisation as part of the FLASH-PT treatment planning process led to higher dose rate coverages for both dose rate definitions considered. FLASH-PT plans generated without a dose rate optimiser significantly improved D_2%_ and target dose homogeneity but were associated with reduced dose rate coverages. Although dose rate optimisation might be useful for increasing dose rate coverage, it should be used with caution as it could significantly degrade the plan quality.

Robust FLASH-PT treatment planning can enhance target dose coverage without compromising doses to OARs or ultra-high dose rates, compared to margin-based planning. However, FLASH-PT plans still do not match target dose coverage, doses to OARs, and dose conformality when compared to their IMPT counterparts. For both nominal and worst-case scenarios, the FLASH-PT plans struggle to achieve IMPT-equivalent D_95%_. Furthermore, robust FLASH-PT plans could only adhere to D_2%_ ≤ 105%, and not D_95%_ ≥ 95%, in the worst-case scenarios.

The dose rate definition is critical when evaluating FLASH-PT plans. The DADR definition led to significantly higher coverages compared to the DR_prct_ definition for both TPSs and optimisation techniques (margin vs. robust) used. Future work will focus on improving D_95%_, reducing OAR doses, and optimising MU/spot delivery to improve plan quality, while further increasing the dose rates.

## Data Availability

The raw data supporting the conclusions of this article will be made available by the authors, without undue reservation.
