# Supplementary material for: The impact of dose rate optimisation and robust optimisation on FLASH proton therapy treatment plan quality and dose rates
Source: Front Oncol. 2025 Dec 19;15:1638319. doi: 10.3389/fonc.2025.1638319 (PMC12757242; doi:10.3389/fonc.2025.1638319)
Supplement: Supplementary file 1 [file DataSheet1.pdf]

```
#####################################################################

# Example MIROpt Configuration File Objectives and Constraints for a Brain Case
# More information can be found on: https://github.com/openFLASH/conformalFLASH/ (accessed 04/09/2025)

#####################################################################

TargetROI: ct_target            # name for target ROI
 ExternalROI: ct_BODY          # name for external ROI - the body contour
 optFunction:
 - name: maxDVH              # Type of objective function. Possible types are: min, max,max_mean, min_mean, minDVH, maxDVH, minDR (minimum average of the percentile dose rate), minDRm (minimum median of the percentile dose rate), minDADRm (minimum median of the dose averaged dose rate)
   ROIname: ct_target         # Name for the ROI to which the objective function needs to be applied
   intersect: ''          # The objective will be applied to "ROIname" intersect with "intersect". Blank if no intersection needed
   remainder: ''          # The objective will be applied to "ROIname" remainder with "remainder". Blank if no remainder needed
   Dref: 10.5              # Reference dose in Gy
   DRref: 0              # Not used
   Vref: 0.02            # Reference volume in 1% only needed for minDVH and maxDVH functions
   impw: 0.5              # Importance weight for the current objective function (impw > 0)
   robust: 0              # Set robust to 1 if robustness optimization needs to be applied to this volume, otherwise set to 0
 - name: minDVH              # Type of objective function. Possible types are: min, max,max_mean, min_mean, minDVH, maxDVH, minDR (minimum average of the percentile dose rate), minDRm (minimum median of the percentile dose rate), minDADRm (minimum median of the dose averaged dose rate)
   ROIname: ct_target         # Name for the ROI to which the objective function needs to be applied
   intersect: ''          # The objective will be applied to "ROIname" intersect with "intersect". Blank if no intersection needed
   remainder: ''          # The objective will be applied to "ROIname" remainder with "remainder". Blank if no remainder needed
   Dref: 10              # Reference dose in Gy
   DRref: 0              # Not used
   Vref: 0.95            # Reference volume in 1% only needed for minDVH and maxDVH functions
   impw: 0.5              # Importance weight for the current objective function (impw > 0)
   robust: 0              # Set robust to 1 if robustness optimization needs to be applied to this volume, otherwise set to 0
 - name: minDVH              # Type of objective function. Possible types are: min, max,max_mean, min_mean, minDVH, maxDVH, minDR (minimum average of the percentile dose rate), minDRm (min$
   ROIname: ct_target         # Name for the ROI to which the objective function needs to be applied
```

```
    intersect: ''          # The objective will be applied to "ROIname" intersect with "intersect". Blank if no intersection needed
    remainder: ''          # The objective will be applied to "ROIname" remainder with "remainder". Blank if no remainder needed
    Dref: 9.5              # Reference dose in Gy#
    DRref: 0               # Not used
    Vref: 1.0              # Reference volume in 1% only needed for minDVH and maxDVH functions
    impw: 0.85             # Importance weight for the current objective function (impw > 0)
    robust: 0              # Set robust to 1 if robustness optimization needs to be applied to this volume, otherwise set to 0
  - name: max              # Type of objective function. Possible types are: min, max,max_mean, min_mean, minDVH, maxDVH, minDR (minimum average of
the percentile dose rate), minDRm (min$
    ROIname: ct_BODY       # Name for the ROI to which the objective function needs to be applied#
    intersect: ''          # The objective will be applied to "ROIname" intersect with "intersect". Blank if no intersection needed
    remainder: ''          # The objective will be applied to "ROIname" remainder with "remainder". Blank if no remainder needed
    Dref: 12               # Reference dose in Gy
    DRref: 0               # Not used
    Vref: 0.95             # Reference volume in 1% only needed for minDVH and maxDVH functions
    impw: 0.5              # Importance weight for the current objective function (impw > 0)
    robust: 0              # Set robust to 1 if robustness optimization needs to be applied to this volume, otherwise set to 0
  - name: minDR            # Type of objective function. Possible types are: min, max,max_mean, min_mean, minDVH, maxDVH, minDR (minimum average of
the percentile dose rate), minDRm (minimum median of the percentile dose rate), minDADRm (minimum median of the dose averaged dose rate
    ROIname: ct_BODY       # Name for the ROI to which the objective function needs to be applied
    intersect: ''          # The objective will be applied to "ROIname" intersect with "intersect". Blank if no intersection needed
    remainder: ''          # The objective will be applied to "ROIname" remainder with "remainder". Blank if no remainder needed
    Dref: 2                # Dose (Gy / FRACTION) in OAR above which the dose rate condition must be respected
    DRref: 40              # Reference dose rate in Gy/s
    Vref: 0.98             # Not used
    impw: 0.00000001       # Importance weight for the current objective function (impw > 0)
    robust: 0              # Set robust to 1 if robustness optimization needs to be applied to this volume, otherwise set to 0
  - name: max              # Type of objective function. Possible types are: min, max,max_mean, min_mean, minDVH, maxDVH, minDR (minimum average of
the percentile dose rate), minDRm (min$
    ROIname: ct_Brain      # Name for the ROI to which the objective function needs to be applied
    intersect: ''          # The objective will be applied to "ROIname" intersect with "intersect". Blank if no intersection needed
    remainder: ''          # The objective will be applied to "ROIname" remainder with "remainder". Blank if no remainder needed
    Dref: 12               # Reference dose in Gy
```

```yaml
    DRref: 0              # Not used
    Vref: 0.95            # Reference volume in 1% only needed for minDVH and maxDVH functions
    impw: 0.5             # Importance weight for the current objective function (impw > 0)
    robust: 0             # Set robust to 1 if robustness optimization needs to be applied to this volume, otherwise set to 0
  - name: minDR           # Type of objective function. Possible types are: min, max,max_mean, min_mean, minDVH, maxDVH, minDR (minimum average of the percentile dose rate), minDRm (minimum median of the percentile dose rate), minDADRm (minimum median of the dose averaged dose rate)
    ROIname: ct_Brain     # Name for the ROI to which the objective function needs to be applied
    intersect: ''         # The objective will be applied to "ROIname" intersect with "intersect". Blank if no intersection needed
    remainder: ''         # The objective will be applied to "ROIname" remainder with "remainder". Blank if no remainder needed#
    Dref: 2               # Dose (Gy / FRACTION) in OAR above which the dose rate condition must be respected
    DRref: 40             # Reference dose rate in Gy/s
    Vref: 0.98            # Not used
    impw: 0.00000001      # Importance weight for the current objective function (impw > 0)
    robust: 0             # Set robust to 1 if robustness optimization needs to be applied to this volume, otherwise set to 0
```

| Function | Constraint | Dose | ROI | Description |
|---|---|---|---|---|
| Physical composite objective | | | | |
| Min dose | | Plan (RBE) | PTVT1_30.0 | Min dose 9.80 Gy (RBE) |
| Max dose | | Plan (RBE) | BODY | Max dose 10.10 Gy (RBE) |
| Max DVH | | Plan (RBE) | Brain | Max DVH 2.00 Gy (RBE) to 2.00% volume |
| Max DVH | | Plan (RBE) | 04_1_Ring_Difference | Max DVH 6.00 Gy (RBE) to 13.00% volume |
| Min DVH | ★ | Plan (RBE) | PTVT1_30.0 | Min DVH 9.90 Gy (RBE) to 100.00% volume |
| Max DVH | ★ | Plan (RBE) | PTVT1_30.0 | Max DVH 10.30 Gy (RBE) to 2.00% volume |
| Min DVH | ★ | Plan (RBE) | PTVT1_30.0 | Min DVH 10.00 Gy (RBE) to 95.00% volume |

*Figure S1: Dose objectives and constraints for a margin-based FLASH-PT plan generated for an example brain case using RayStation.*

| Function | Constraint | Dose | ROI | Description | Robust |
|---|---|---|---|---|---|
| Physical composite objective | | | | | |
| Max DVH | | Plan (RBE) | GTVT1_30.0 | Max DVH 10.50 Gy (RBE) to 2.00% volume | ★ |
| Min DVH | | Plan (RBE) | GTVT1_30.0 | Min DVH 9.50 Gy (RBE) to 95.00% volume | ★ |
| Min DVH | | Plan (RBE) | GTVT1_30.0 | Min DVH 10.00 Gy (RBE) to 95.00% volume | ★ |
| Max dose | | Plan (RBE) | Brain | Max dose 10.60 Gy (RBE) | |
| Max dose | | Plan (RBE) | BODY | Max dose 10.50 Gy (RBE) | |
| Max DVH | | Plan (RBE) | 04_1_Ring_Difference | Max DVH 8.00 Gy (RBE) to 3.00% volume | |

*Figure S2: Dose objectives and constraints for a robust FLASH-PT plan generated for an example brain case using RayStation.*

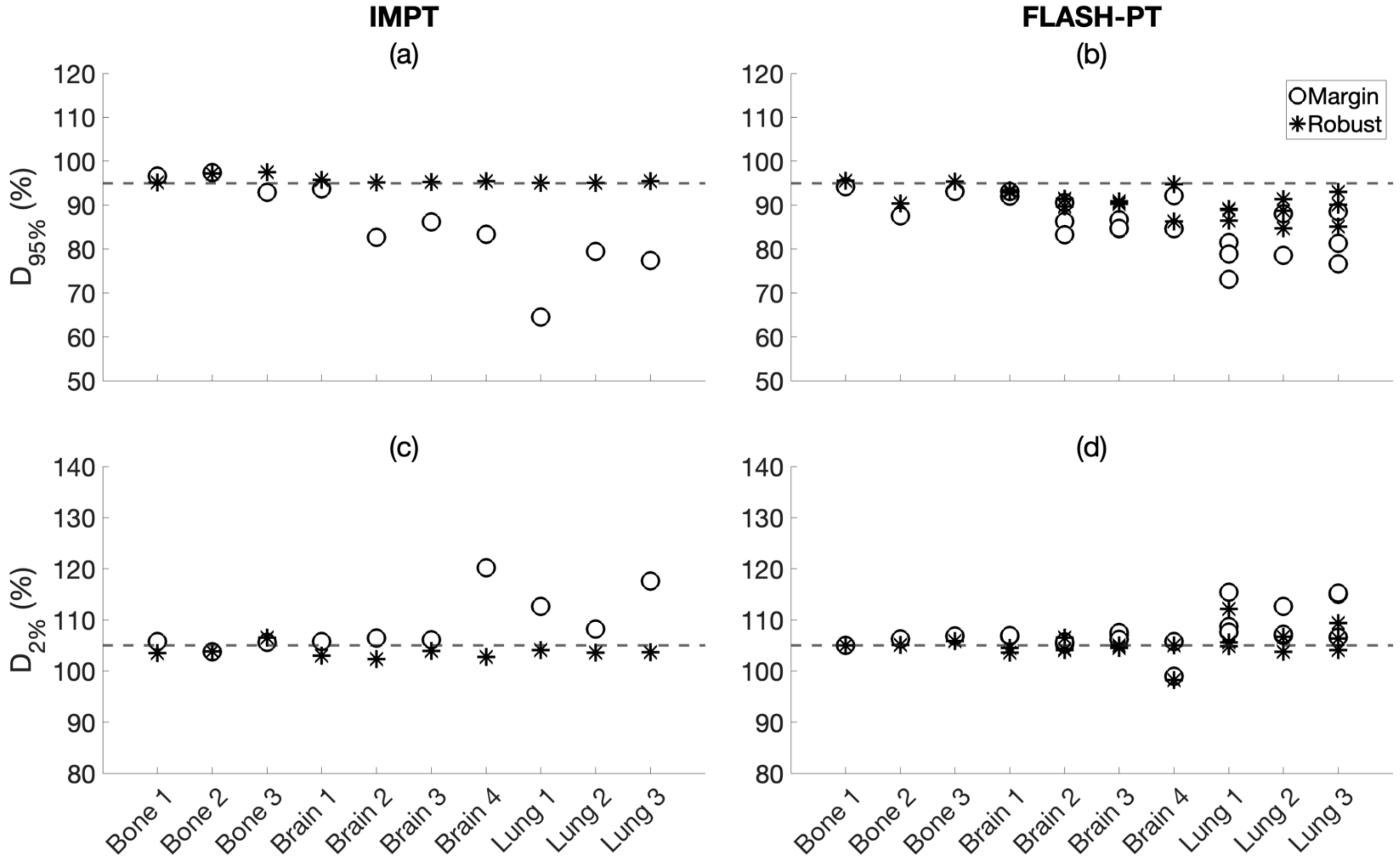


*Figure S3: Worst-case scenario comparisons between margin (o) and robust (*) optimisation techniques for intensity modulated proton therapy (IMPT) and Bragg peak FLASH proton therapy (FLASH-PT). The worst-case scenarios were evaluated for $D_{95\%}$ and $D_{2\%}$ of the target volumes and are shown for each individual patient case for both IMPT and FLASH-PT, and for each individual fraction for FLASH-PT. Grey dashed lines indicate target dose limits of $D_{95\%}$ = 95% for (a) and (b), and $D_{2\%}$ = 105% for (c) and (d).*

| Cases Considered | Margin WC $D_{95\%}$ (%) | Robust WC $D_{95\%}$ (%) | Margin WC $D_{2\%}$ (%) | Robust WC $D_{2\%}$ (%) |
|---|---|---|---|---|
| **All** | 86.50 | 90.64 | 106.89 | 105.00 |
| **Bone** | 93.13 | 95.38 | 106.25 | 105.13 |
| **Brain** | 86.50 | 91.10 | 105.98 | 104.50 |
| **Lung** | 81.27 | 88.93 | 108.73 | 106.33 |

*Table S1: Median values of the worst-case (WC) scenario $D_{95\%}$ (%) and $D_{2\%}$ (%) for margin-based and robust Bragg peak FLASH proton therapy treatment plans, shown for all patient cases, as well as for the individual target sites.*

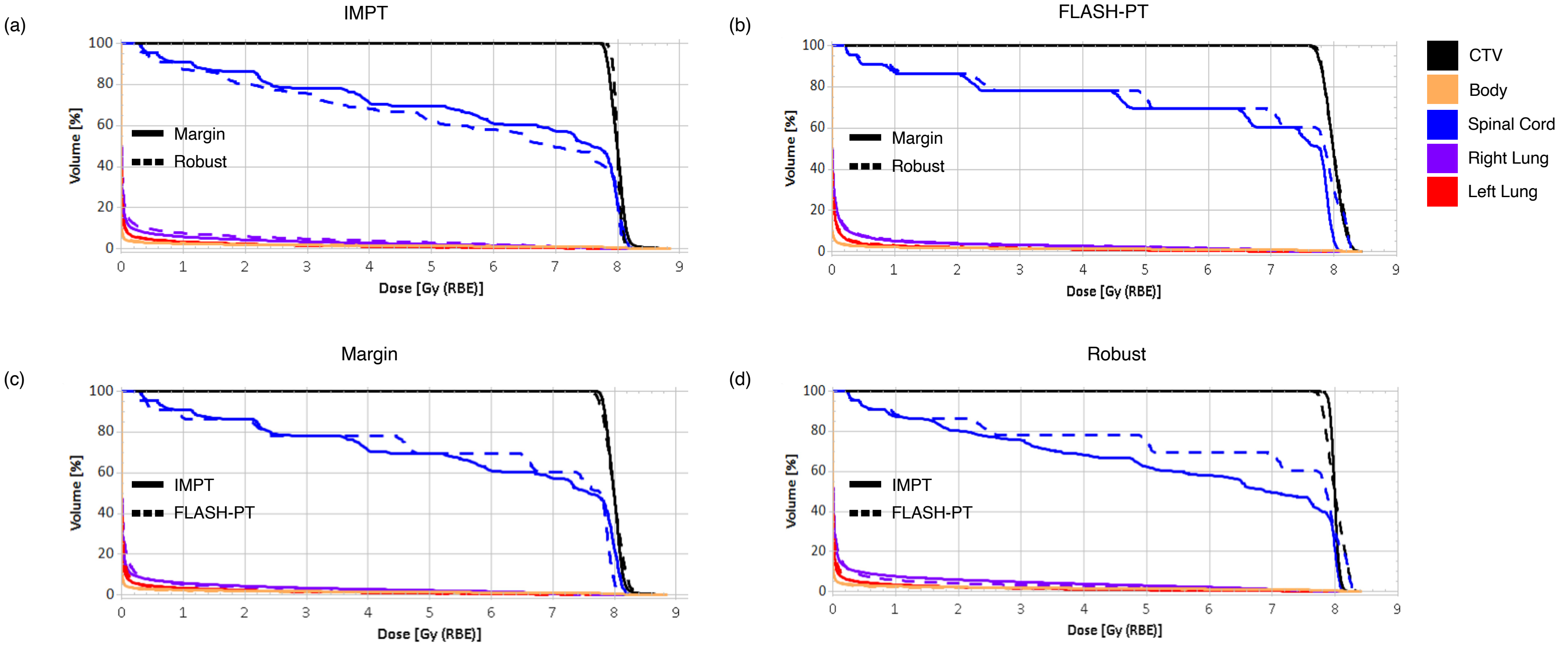


*Figure S4: Dose volume histograms (DVHs) for RayStation plans simulated for an example bone case (Bone 1). Treatment techniques (margin vs. robust) are compared for both (a) IMPT and (b) FLASH-PT plans. Comparisons of IMPT and FLASH-PT plans are made for (c) margin-based and (d) robust plans. Treatment planning system comparisons could not be made for any bone case as MIROpt plans could not be generated due to target size constraints.*

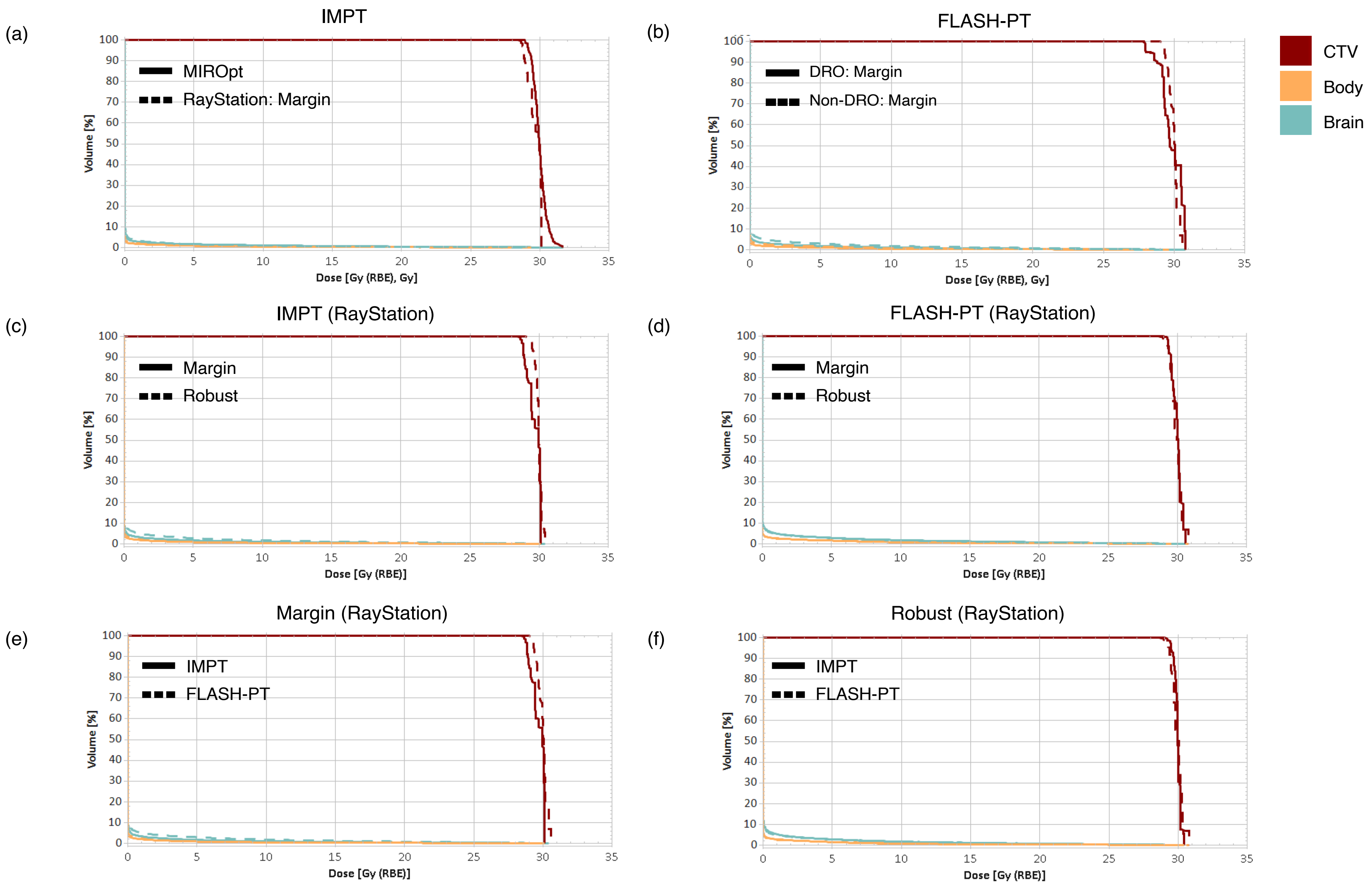


*Figure S5: Dose volume histograms (DVHs) for an example brain case (Brain 2). Treatment planning system comparison (MIROpt vs. RayStation) is made for (a) IMPT plans, with (b) comparisons between dose rate optimised (DRO) and non-dose rate optimised (non-DRO) FLASH-PT, treatment techniques (margin vs. robust) are then compared for both (c) IMPT and (d) FLASH-PT plans simulated using RayStation. Comparisons between IMPT and FLASH-PT plans are then shown for both (e) margin-based and (f) robust IMPT and FLASH-PT plans.*

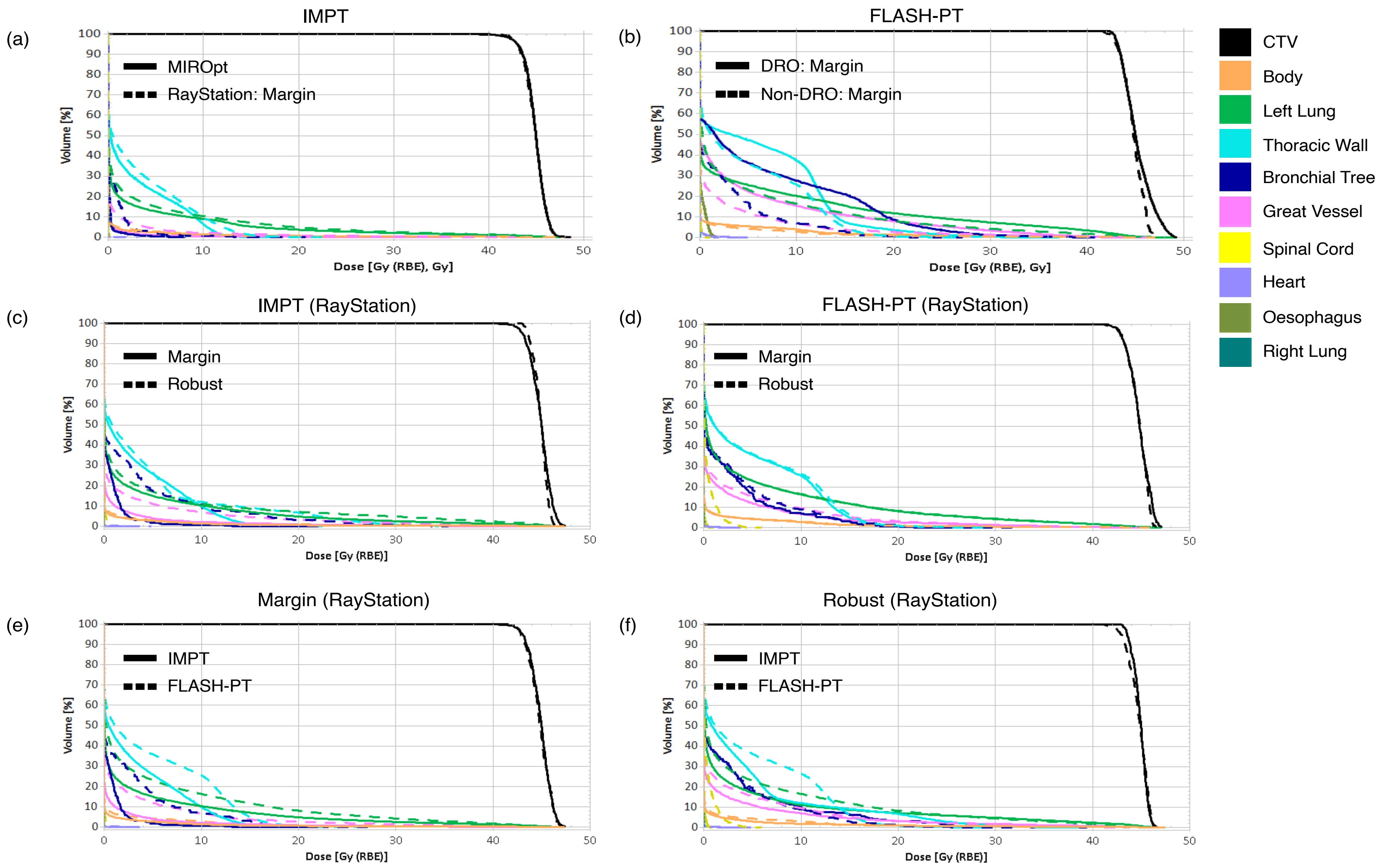


*Figure S6: Dose volume histograms (DVHs) for an example lung case (Lung 2). Treatment planning system comparison (MIROpt vs. RayStation) is made for (a) IMPT plans, with (b) comparisons between dose rate optimised (DRO) and non-dose rate optimised (non-DRO) FLASH-PT, treatment techniques (margin vs. robust) are then compared for both (c) IMPT and (d) FLASH-PT plans simulated using RayStation. Comparisons between IMPT and FLASH-PT plans are then shown for both (e) margin-based and (f) robust IMPT and FLASH-PT plans.*

*Table S2: Intensity modulated proton therapy doses to the spinal cord for the different bone cases (Bone 1, Bone 2, Bone 3) and treatment planning techniques (margin-based and robust optimisation), generated using RayStation. The dose protocol used for these cases is RTOG 0631 [39].*

| **Patient** | | | **Bone 1** | | **Bone 2** | | **Bone 3** | |
|---|---|---|---|---|---|---|---|---|
| **Technique** | | | **Margin** | **Robust** | **Margin** | **Robust** | **Margin** | **Robust** |
| **Protocol** | **OAR** | **Constraint** | | | | | | |
| RTOG 0631 | Spinal Cord | 10 Gy < 10% volume (%) | 0 | 0 | 0 | 0 | 0 | 0 |
| RTOG 0631 | Spinal Cord | Max 10 Gy (D1%) (Gy) | 8.2 | 8.11 | 8.2 | 8.28 | 8.06 | 8.09 |

*Table S3: Bragg peak FLASH proton therapy doses to the spinal cord for the different bone cases (Bone 1, Bone 2, Bone 3) and treatment planning techniques (margin-based and robust optimisation), generated using RayStation. The dose protocol used for these cases is RTOG 0631 [39].*

| **Patient** | | | **Bone 1** | | **Bone 2** | | **Bone 3** | |
|---|---|---|---|---|---|---|---|---|
| **Technique** | | | **Margin** | **Robust** | **Margin** | **Robust** | **Margin** | **Robust** |
| **Protocol** | **OAR** | **Constraint** | | | | | | |
| RTOG 0631 | Spinal Cord | 10 Gy < 10% volume (%) | 0 | 0 | 0 | 0 | 0 | 0 |
| RTOG 0631 | Spinal Cord | Max 10 Gy (D1%) (Gy) | 8.07 | 8.28 | 8.52 | 8.77 | 8.47 | 8.27 |

*Table S4: Intensity modulated proton therapy doses to organs at risk (OAR) for the different brain cases (Brain 1, Brain 2, Brain 3, Brain 4), dose rate optimisations (dose rate optimised (DRO) and non-dose rate optimised (non-DRO)), and treatment planning techniques (margin-based and robust optimisation). Protocols used are from CORSAIR [37]. OAR dose constraints are given for each OAR, protocol, patient case, and treatment planning system/technique. Dashes (-) indicate that the specific OAR/constraint did not apply to that specific patient case. Brain 1 and Brain 4 were palliative and so the target volumes both exceeded 20 cm3; the first constraint could therefore not be applied to these cases. The Brain Stem was not contoured for Brain 4 due to complete overlap with the CTV; for Brain 2 and Brain 3 the target was not located near the Brain Stem and so it was not applicable as an OAR.*

| **Patient** | | | **Brain 1** | | | **Brain 2** | | | **Brain 3** | | | **Brain 4** | | |
|---|---|---|---|---|---|---|---|---|---|---|---|---|---|---|
| **System and Technique** | | | **DRO: Margin** | **Non-DRO: Margin** | **Non-DRO: Robust** | **DRO: Margin** | **Non-DRO: Margin** | **Non-DRO: Robust** | **DRO: Margin** | **Non-DRO: Margin** | **Non-DRO: Robust** | **DRO: Margin** | **Non-DRO: Margin** | **Non-DRO: Robust** |
| **Protocol** | **OAR** | **Constraint** | | | | | | | | | | | | |
| CORSAIR | Brain + target volume | D20cm$^3$ < 20 Gy (Gy) (optimal) | Target volume > 20 cm$^3$ | Target volume > 20 cm$^3$ | Target volume > 20 cm$^3$ | 8.45 | 7.52 | 7.84 | 4.96 | 5.97 | 7.85 | Target volume > 20 cm$^3$ | Target volume > 20 cm$^3$ | Target volume > 20 cm$^3$ |
| CORSAIR | Brain stem | $D_{max}$(0.035) < 18 Gy (Gy) (optimal) | - | - | - | - | - | - | - | - | - | 0.00 | 0.00 | 0.00 |

*Table S5: Bragg peak FLASH proton therapy doses to organs at risk (OAR) for the different brain cases (Brain 1, Brain 2, Brain 3, Brain 4), dose rate optimisations (dose rate optimised (DRO) and non-dose rate optimised (non-DRO)), and treatment planning techniques (margin-based and robust optimisation). Protocols used are from CORSAIR [37]. OAR dose constraints are given for each OAR, protocol, patient case, and treatment planning system/technique. Dashes (-) indicate that the specific OAR/constraint did not apply to that specific patient case. Brain 1 and Brain 4 were palliative and so the target volumes both exceeded 20 cm3; the first constraint could therefore not be applied to these cases. The Brain Stem was not contoured for Brain 4 due to complete overlap with the CTV; for Brain 2 and Brain 3 the target was not located near the Brain Stem and so it was not applicable as an OAR.*

| **Patient** | | | **Brain 1** | | | **Brain 2** | | | **Brain 3** | | | **Brain 4** | | |
|---|---|---|---|---|---|---|---|---|---|---|---|---|---|---|
| **System and Technique** | | | **DRO: Margin** | **Non-DRO: Margin** | **Non-DRO: Robust** | **DRO: Margin** | **Non-DRO: Margin** | **Non-DRO: Robust** | **DRO: Margin** | **Non-DRO: Margin** | **Non-DRO: Robust** | **DRO: Margin** | **Non-DRO: Margin** | **Non-DRO: Robust** |
| **Protocol** | **OAR** | **Constraint** | | | | | | | | | | | | |
| CORSAIR | Brain + target volume | D20cm$^3$ < 20 Gy (Gy) (optimal) | Target volume > 20 cm$^3$ | Target volume > 20 cm$^3$ | Target volume > 20 cm$^3$ | 8.77 | 14.81 | 14.08 | 5.12 | 6.79 | 12.26 | Target volume > 20 cm$^3$ | Target volume > 20 cm$^3$ | Target volume > 20 cm$^3$ |
| CORSAIR | Brain stem | $D_{max}(0.035)$ < 18 Gy (Gy) (optimal) | - | - | - | - | - | - | - | - | - | 0.00 | 0.00 | 0.00 |

*Table S6: Intensity modulated proton therapy doses to organs at risk (OAR) for the different lung cases (Lung 1, Lung 2, Lung 3), dose rate optimisations (dose rate optimised (DRO) and non-dose rate optimised (non-DRO)), and treatment planning techniques (margin-based and robust optimisation). Protocols used are the UK Consensus on Normal Tissue Dose Constraints for Stereotactic Radiotherapy and CORSAIR [37,38]. OAR dose constraints are given for each OAR, protocol, patient case, and treatment planning system/technique. Dashes (-) indicate that the specific OAR/constraint did not apply to that specific patient case. For Lung 1, the target was not located near the Great Vessel or the Bronchial Tree and so these were not applicable as OARs.*

| **Patient** | | | **Lung 1** | | | **Lung 2** | | | **Lung 3** | | |
|---|---|---|---|---|---|---|---|---|---|---|---|
| **System and Technique** | | | **DRO: Margin** | **Non-DRO: Margin** | **Non-DRO: Robust** | **DRO: Margin** | **Non-DRO: Margin** | **Non-DRO: Robust** | **DRO: Margin** | **Non-DRO: Margin** | **Non-DRO: Robust** |
| **Protocol** | **OAR** | **Constraint** | | | | | | | | | |
| 3 fx UK Consensus (Stereotactic) | Heart | $D_{max}$ (0.5 $cm^3$) < 24 Gy (Gy) (optimal) | 0.08 | 0.12 | 0.29 | 0.06 | 0.11 | 0.99 | 4.99 | 8.04 | 11.78 |
| 3 fx UK Consensus (Stereotactic) | Great Vessel | $D_{max}$ (0.5$cm^3$) < 45 Gy (Gy) (mandatory) | - | - | - | 6.69 | 22.97 | 39,16 | 25.2 | 36.91 | 43.51 |
| CORSAIR | Great Vessel | D10$cm^3$ < 39 Gy (Gy) (optimal) | | | | | | | | | |
| CORSAIR | Lung | $D_{mean}$ < 8 Gy (Gy) (optimal) | - | - | - | 0.09 | 0.66 | 4.1 | 2.74 | 5.8 | 12.38 |
| CORSAIR | Lung | V20 Gy < 15% (%) (mandatory) | 2.03 | 2.69 | 4.06 | 2.44 | 2.99 | 3.82 | 2.02 | 2.48 | 3.09 |
| CORSAIR | Lung | V20 Gy < 10% (%) (optimal) | 2.88 | 4.05 | 6.76 | 3.58 | 4.74 | 7.05 | 5.8 | 4.03 | 5.01 |
| CORSAIR | Spinal Cord | $D_{max}$(0.1 $cm^3$) < 18 Gy (Gy) (optimal) | 2.88 | 4.05 | 6.76 | 3.58 | 4.74 | 7.05 | 5.8 | 4.03 | 5.01 |
| 3 fx UK Consensus (Stereotactic) | Bronchial Tree | $D_{max}$(0.5 $cm^3$) < 30 Gy (Gy) (optimal) | 0 | 0 | 0 | 0.09 | 0.05 | 0.47 | 0 | 0 | 0 |

*Table S7: Bragg peak FLASH proton therapy doses to organs at risk (OAR) for the different lung cases (Lung 1, Lung 2, Lung 3), dose rate optimisations (dose rate optimised (DRO) and non-dose rate optimised (non-DRO)), and treatment planning techniques (margin-based and robust optimisation). Protocols used are the UK Consensus on Normal Tissue Dose Constraints for Stereotactic Radiotherapy and CORSAIR [37,38]. OAR dose constraints are given for each OAR, protocol, patient case, and treatment planning system/technique. Dashes (-) indicate that the specific OAR/constraint did not apply to that specific patient case. For Lung 1, the target was not located near the Great Vessel or the Bronchial Tree and so these were not applicable as OARs*. Bold values indicate that they exceeded the optimal dose constraint, whereas bold and underlined values indicate that they exceeded the mandatory dose constraints.

| **Patient** | | | **Lung 1** | | | **Lung 2** | | | **Lung 3** | | |
|---|---|---|---|---|---|---|---|---|---|---|---|
| **System and Technique** | | | **DRO: Margin** | **Non-DRO: Margin** | **Non-DRO: Robust** | **DRO: Margin** | **Non-DRO: Margin** | **Non-DRO: Robust** | **DRO: Margin** | **Non-DRO: Margin** | **Non-DRO: Robust** |
| **Protocol** | **OAR** | **Constraint** | | | | | | | | | |
| 3 fx UK Consensus (Stereotactic) | Heart | $D_{max}$ (0.5 $cm^3$) < 24 Gy (Gy) (optimal) | 0.01 | 0.34 | 0.38 | 2.66 | 1.31 | 1.71 | 20.29 | 17.79 | 17.19 |
| 3 fx UK Consensus (Stereotactic) | Great Vessel | $D_{max}$ (0.5$cm^3$) < 45 Gy (Gy) (mandatory) | - | - | - | 38.98 | 35.33 | 34.46 | **45.29** | 40.02 | 40.83 |
| CORSAIR | Great Vessel | D10$cm^3$ < 39 Gy (Gy) (optimal) | - | - | - | 13.03 | 4.81 | 7.43 | 29.05 | 22.71 | 27.57 |
| CORSAIR | Lung | $D_{mean}$ < 8 Gy (Gy) (optimal) | 3.82 | 3.93 | 4.52 | 5.73 | 4.67 | 4.73 | 2.88 | 3.54 | 3.59 |
| CORSAIR | Lung | V20 Gy < 15% (%) (mandatory) | 6.72 | 5.81 | 6.58 | 11.66 | 7.98 | 8.22 | 5.01 | 5.95 | 5.51 |
| CORSAIR | Lung | V20 Gy < 10% (%) (optimal) | 6.72 | 5.81 | 6.58 | 11.66 | 7.98 | 8.22 | 5.01 | 5.95 | 5.51 |
| CORSAIR | Spinal Cord | $D_{max}$(0.1 $cm^3$) < 18 Gy (Gy) (optimal) | 0 | 0 | 0 | 0 | 0.41 | 3.86 | 0 | 0 | 0 |
| 3 fx UK Consensus (Stereotactic) | Bronchial Tree | $D_{max}$(0.5 $cm^3$) < 30 Gy (Gy) (optimal) | - | - | - | 18.94 | 6.51 | 8.23 | 0.01 | 3.84 | 3.72 |
